# Fas Signaling in Dendritic Cells Mediates Th2 Polarization in HDM-Induced Allergic Pulmonary Inflammation

**DOI:** 10.3389/fimmu.2018.03045

**Published:** 2018-12-21

**Authors:** Miaomiao Han, Ran Hu, Jingyu Ma, Baohua Zhang, Ce Chen, Huabin Li, Jun Yang, Gonghua Huang

**Affiliations:** ^1^Department of Otolaryngology-Head and Neck Surgery, Center for Allergic and Inflammatory Diseases, Affiliated Eye and ENT Hospital, Fudan University, Shanghai, China; ^2^Shanghai Institute of Immunology, Shanghai Jiao Tong University School of Medicine, Shanghai, China; ^3^Xin Hua Hospital Affiliated to Shanghai Jiao Tong University School of Medicine, Shanghai, China; ^4^Guangdong Provincial Key Laboratory of Medical Molecular Diagnostics, Guangdong Medical University, Dongguan, China

**Keywords:** allergic inflammation, dendritic cells, Fas, house dust mite, Th2

## Abstract

Fas–Fas ligand (FasL) signaling plays an important role in the development of allergic inflammation, but the cellular and molecular mechanisms are still not well known. By using the bone marrow-derived dendritic cell (BMDC) transfer-induced pulmonary inflammation model, we found that house dust mite (HDM)-stimulated FAS-deficient BMDCs induced higher Th2-mediated allergic inflammation, associated with increased mucus production and eosinophilic inflammation. Moreover, FAS-deficient BMDCs promoted Th2 cell differentiation upon HDM stimulation *in vitro*. Compared to wild-type BMDCs, the Fas-deficient BMDCs had increased ERK activity and decreased IL-12 production upon HDM stimulation. Inhibition of ERK activity could largely increase IL-12 production, consequently restored the increased Th2 cytokine expression of OT-II CD4^+^ T cells activated by Fas-deficient BMDCs. Thus, our results uncover an important role of DC-specific Fas signaling in Th2 differentiation and allergic inflammation, and modulation of Fas signaling in DCs may offer a useful strategy for the treatment of allergic inflammatory diseases.

## Introduction

Allergic inflammation has been generally considered as a T helper (Th) 2-mediated chronic immune response (1). Th2 cells produce effector cytokines such as IL-4, IL-5, and IL-13 to mediate the respiratory symptoms. Among these effector cytokines, IL-4 is involved in IgE synthesis and IL-5 can drive eosinophilia in lung tissue, while IL-13 contributes to mucus overproduction, airway hyper-responsiveness (AHR), goblet cell metaplasia and airway remodeling (1–3). House dust mite (HDM) has been reported to cause 50–85% of allergic asthmatic inflammation (4). The polarization of a Th2-mediated immune response to inhaled allergens (such as HDM) is determined by the status of dendritic cells (DCs). DCs promote Th2 differentiation through upregulation of the expression of several costimulatory molecules such as CD86, OX40L and polarization cytokines including IL-6, IL-10, and IL-33 (5–8), as well as Th2-cell-attracting chemokines, such as CCL17 and CCL22 (9). Although accumulating evidence suggests that DCs are sufficient and necessary to initiate Th2 responses, the underlying signaling mechanism for DCs to direct Th2 differentiation and function is still not well-understood.

Fas (CD95, also named APO-1) signaling is widely considered to mediate apoptosis upon binding to its ligand (FasL, also called “CD95L or APO-1L”) or its agonist antibody (10). In an ovalbumin (OVA)-induced mouse asthma model, Fas-deficient mice have delayed resolution of airway hyperresponsiveness (AHR) compared to wild-type mice (11). Further study indicates that Fas deficiency in T cells contributes to the prolonged resolution of airway inflammation (12). Recent studies have shown that Fas–FasL interaction could also activate non-apoptotic pathways, such as Fas signaling leading to T cell activation, proliferation and differentiation (13) and promoting Th17 polarization and Th17-mediated autoimmunity (14). Fas-deficient mice sensitized with OVA increase the expression of IL-4, IL-5, and IL-13 compared to wild-type mice (15). Although DCs are pivotal in regulating T cell activation, proliferation, differentiation and allergic inflammation, the role of Fas signaling in DCs in driving Th2 differentiation and Th2-mediated allergic inflammation still need to be elucidated.

In the present study, we used the well-established model by adoptively transferring HDM-pulsed BMDCs to recipient mice to explore the role of FAS signaling in DCs in pulmonary inflammation. We found that Fas deficiency in DCs led to increased mucus production, eosinophilic inflammation and Th2 response *in vivo*. Fas-deficient BMDCs promoted the production of Th2-related cytokines such as IL-4 and IL-13. Further mechanistic study showed that DCs directed Th2 differentiation by modulating the Fas–ERK–IL-12 axis. Collectively, our results identify an important signaling mechanism of DC-mediated Th2 responses and modulation of Fas signaling in DCs might offer a useful strategy for the treatment of eosinophilic lung inflammatory diseases.

## Materials and Methods

### Mice

B6.MRL-*Tnfrsf6*^lpr^ mice were from The Jackson Laboratory. C57BL/6 mice were from Shanghai SLAC Laboratory Animal Center (Shanghai, China). All mice were kept in a specific pathogen-free (SPF) barrier facility maintained by Shanghai Jiao Tong University School of Medicine. All the experimental mice were used at 6–10 weeks. Animal protocols were approved by Institutional Animal Care and Use Committee of Shanghai Jiao Tong University School of Medicine.

### BMDC Culture

BMDCs were cultured as previously described (16). Briefly, bone marrow cells were collected by perfusing mouse femur and tibia. After red blood cell lysis, cells were cultured in RPMI 1640 (Invitrogen Corp.) supplemented with 10% fetal calf serum (FCS, Gibco), penicillin and streptomycin (Invitrogen), 2-mercaptoethanol (Sigma-Aldrich), supernatant from J5 cells (provided by Dr. Qibin Leng, Institut Pasteur of Shanghai, China) expressing GM-CSF (1:50) and 10 ng/ml IL-4 (R&D). On day 3, the entire medium was removed and replaced with fresh differentiation medium. On day 7, the cells were harvested for analyses. The purity of CD11c^+^ BMDCs was >80%.

### BMDC Adoptive Transfer Experiment

In BMDC adoptive transfer experiment, wild-type or Fas-deficient BMDCs were pulsed with 50 μg/ml HDM (Greer Laboratories, Lenoir, NC) for 12 h, washed, and then 1 × 10^6^ HDM-pulsed BMDCs were administered intravenously into naïve C57BL/6 recipients. Recipients transferred with un-pulsed wild-type or Fas-deficient BMDCs as control. On day 10–12, all the recipients were lightly anesthetized and challenged intranasally with 10 μg HDM in 40 μl PBS and the mice were sacrificed for analysis on day 13.

### Antibodies and Flow Cytometry

Anti-mouse CD11c (N418), MHC-II (M5/114.15.2), CD11b (M1/70), Ly6G (RB6-8C5), siglec F (E50-2440), CD4 (RM4-5), TCRβ (H57-597), IL-4 (11B11), IL-13 (eBio13A), IL-17A (eBio17B7), CD25 (PC61.5), CD44 (IM7), CD62L (MEL-14), CD69 (H1.2F3), Ki-67 (SolA15), IL-12p40 (C17.8), CD45 (30-F11), CD178 (MFL3) antibodies were obtained from eBioscience. Anti-mouse IL-5 (TRFK5) was obtained from BD Biosciences. Anti-mouse CCR3 (J073E5) was obtained from Biolegend. For surface staining, cells were stained with antibodies in PBS containing 1% FCS (Hyclone) on ice for 30 min. For intracellular staining, cells were stimulated with PMA (Sigma-Aldrich) and ionomycin (Sigma-Aldrich) for 5 h in the presence of Golgistop (BD Biosciences) before being stained according to the manufacturer's instructions (eBioscience). CD4^+^T cell proliferation was detected by anti-Ki-67 staining according to the manufacturer's instructions (eBioscience) 3 days after co-culture. Labeling OTII CD4^+^ T cells with CFSE (carboxyfluorescein diacetate succinimidyl diester; Invitrogen), cell proliferation was detected by flow cytometry 4 days after co-culture. For cell apoptosis analysis, cells were stained with CaspACE™ FITC-VAD-FMK *in situ* Marker (Promega). The samples were acquired on a FACSCantoII (BD) or LSRFortessa^TM^ X-20 (BD) and analyzed with FlowJo software (Treestar).

### Bronchoalveolar Lavage Fluid (BALF) Collection and Lung Mononuclear Cell Isolation

For BALF collection, lung tissues were lavaged with 1 ml cold PBS for 3 times and the supernatant was collected. Lung mononuclear cells were prepared as previously described (17). Briefly, lung tissues were removed, minced and digested with 1 mg/ml Collagenase IV (Life Technologies) in RPMI-1640 (Hyclone) with 5% FCS (Hyclone) for 45 min at 37°C. Cells were enriched by using 38% Percoll gradient (GE Healthcare Life Sciences). Red blood cells were lyzed with ACK lysis buffer (R&D Systems). Cells were harvested for analyses.

### Histology

Left lobe of lung tissues were removed from mice after BALF collection, fixed with 4% paraformaldehyde (PFA) at room temperature for 24 h and embedded in paraffin, cut into 5-μm sections for hematoxylin and eosin (HE) or periodic acid-Schiff (PAS) staining. The lung inflammation was blindly quantified using HE-stained sections according to the criteria previously published (18). The quantification of the goblet cell hyperplasia in the airway was done as previously described (19).

### RNA Isolation and Quantitative PCR (qPCR)

Total RNAs of lung tissues were isolated using Trizol regent (Invitrogen). Total RNAs of cells were isolated using RNeasy mini Kit (QIAGEN) according to the manufacturer's instructions. 1 μg total RNA was used for reverse transcription with PrimeScript RT Master Mix (TAKARA) according to the manufacturer's instructions in a total volume of 20 μl. qPCR was carried out with SYBR Green PCR Master Mix (Applied Biosystems) in a Vii7 Real-Time PCR system (Applied Biosystems). mRNA expression of genes was normalized to *Hprt*. The primers shown below were from Primerbank: *Hprt*, forward primer: TCAGTCAACGGGGGACATAAA, reverse primer: GGGGCTGTACTGCTTAACCAG; *Il4*, forward primer: GGTCTCAACCC CCAGCTAGT, reverse primer: GCCGATGATCTCTCTCAAGTGAT; *Il17a*, forward primer: TCAGCGTGTCCAAACACTGAG, reverse primer: CGCCAAGGGAG TTAAAGACTT; *Ifng*, forward primer: GCCACGGCACAGTCATTGA, reverse primer: TGCTGATGGCCTGATTGTCTT; *Il6*, forward primer: CTGCAAGAGACTTCCATCCAG, reverse primer: AGTGGTATAGACAGGTATGTTGG; *Il12p35*, forward primer: CAATCACGCTACCTCCTCTTT, reverse primer: CAGCAGTGCAGGAATAATGTTTC; *Il12p40*, forward primer: GTCCTCAGAAGCTAACCATCTC, reverse primer: CCAGAGC CTATGACTCCATGTC; *Il10*, forward primer: CTTACTGACTGGCATGAGGATCA, reverse prime: GCAGCTCTAGGAGCATGTGG. The other primers were used as described, such as *Il5, Il13* (20), *Gata3* (21), *Il9* (22), *Tnfsf4* (8), *Cd86* (23), *Tslp* (24).

### Cell Stimulation and Culture

BMDCs were stimulated with HDM in the presence or absence of Fas agonistic antibody Jo2 (1 μg/ml, BD) or Fas antagonistic antibody kp7-6 (1 mM, Merk) for 5 h for RNA analysis. For drug inhibitor treatments, cells were incubated with vehicle (DMSO) or U0126 (10 μM) (from Calbiochem) for 0.5–1 h before adding other stimuli. For BMDC–T cell co-culture, BMDCs and flow cytometry-sorted naïve OT-II CD4^+^ T cells (CD4^+^25^−^CD44^−^CD62L^+^, purity >99%) were mixed at a ratio of 1:10 in the presence of OVA_323−339_ peptide and HDM, and then the CD4^+^ T cells were harvested at 48 h for mRNA analysis or supernatant was harvested at 72 h for ELISA. For cytokine treatment, cultures were supplemented with 1 ng/ml IL-12p70 (R&D).

### Protein Analysis

Concentrations of IL-4 and IL-13 were measured by ELISA according to the manufacturer's instructions (R&D; eBioscience). Read the OD values at 450 nm on the MultiSKAN GO microplate reader (Thermo). Immunoblot analysis was performed as described (25) with antibody to ERK phosphorylated at Thr202 and Tyr204, antibody to p38 phosphorylated at Thr180 and Tyr182, antibody to JNK phosphorylated at Thr183 and Tyr185 and antibody to ERK (all from Cell Signaling Technology), antibody to alpha tubulin (Proteintech).

### Statistical Analysis

All statistical analyses were performed by unpaired Student's *t-*tests or ANOVA using GraphPad Prism software (version 5.0). *P* < 0.05 was considered significant. Results represent means ± SEM.

## Results

### Fas-Deficient BMDCs Enhance HDM-Induced Pulmonary Inflammation

**Figure 1:**
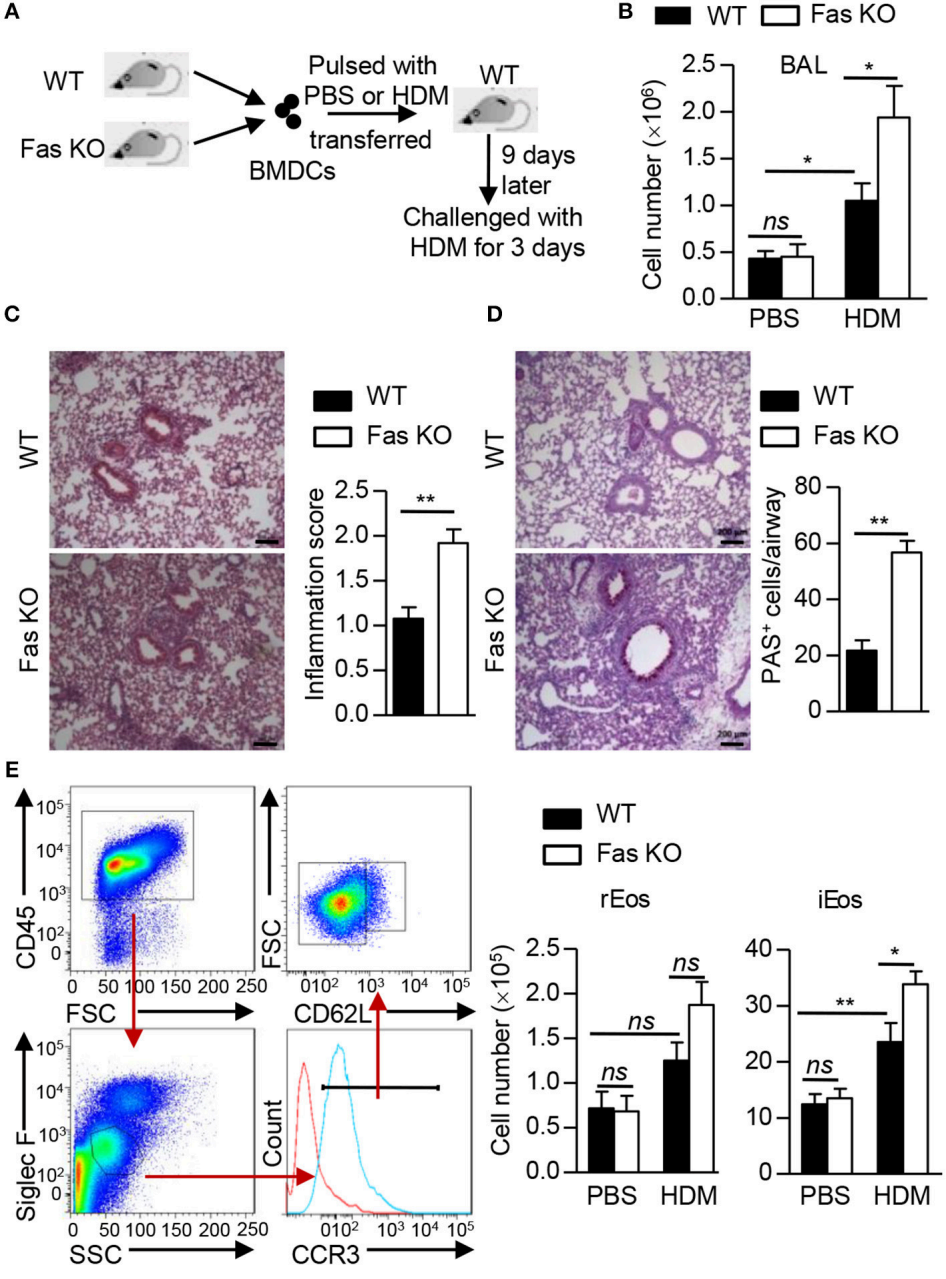
Fas-deficient BMDCs enhance HDM-induced pulmonary inflammation. **(A)** Protocol for HDM-induced allergic airway inflammation using BMDC transfer experiment. **(B)** Total cell number in the BAL. **(C)** HE staining of lung sections from recipients transferred with HDM-pulsed wild-type or Fas-deficient BMDCs and quantitative analysis. Scale Bars represent 200 μm. **(D)** PAS staining of lung sections from recipients transferred with HDM-pulsed wild-type or Fas-deficient BMDCs and quantification of PAS^+^ cells in airways. Scale Bars represent 200 μm. **(E)** Gating strategy and cell number of inflammatory eosinophils (iEos) (CD45^+^SiglecF^int^CCR3^+^CD62L^−^) and resident eosinophils (rEos) (CD45^+^SiglecF^int^CCR3^+^CD62L^+^) in lung tissues of recipients transferred with wild-type or Fas-deficient BMDCs pulsed or un-pulsed with HDM. ^*^P < 0.05, ^**^P < 0.01, *ns*, not significant. Data are representative of two independent experiments with 5–8 mice per group **(B–E)**. Student's *t*-tests **(C,D)** or two-way ANOVA **(B,E)** were performed and data were presented as mean ± SEM.

To explore the role of Fas signaling in DCs in the regulation of HDM-induced allergic inflammation in mice, we used a BMDC adoptive transfer protocol to induce lung inflammation (Figure 1A). We transferred HDM-pulsed or un-pulsed wild-type or Fas-deficient BMDCs into naïve wild-type recipient mice. After HDM re-challenged, mice received HDM-pulsed BMDCs showed higher total cell number in the bronchoalveolar lavage (BAL) compared to mice received un-pulsed BMDCs (Figure 1B). A significantly increased total cell number was also observed in the BAL of recipients transferred with HDM-pulsed Fas-deficient BMDCs (Figure 1B). We also observed higher inflammatory cell infiltration and mucus production in lung tissues of recipients transferred with HDM-pulsed Fas-deficient BMDCs than those transferred with HDM-pulsed wild-type BMDCs (Figures 1C,D). Flow cytometry showed that a dramatically increased eosinophil infiltration both in the BAL and lung tissues of recipients transferred with HDM-pulsed Fas-deficient BMDCs compared to those transferred with HDM-pulsed wild-type BMDCs (Supplementary Figures 1A,B). We also analyzed the inflammatory eosinophils (iEos) (CD45^+^Siglec F^int^CCR3^+^CD62L^−^) and resident eosinophils (rEos) (CD45^+^Siglec F^int^CCR3^+^CD62L^+^) in lung tissues (26). The cell number of iEos was increased in recipients transferred with HDM-pulsed Fas-deficient BMDCs compared to those transferred with HDM-pulsed wild-type BMDCs, but rEos was comparable between recipients transferred with HDM-pulsed wild-type BMDCs and those transferred with Fas-deficient BMDCs (Figure 1E). However, the neutrophil infiltration had no difference between the two groups (Supplementary Figures 1C,D). Together, these data indicate that HDM-pulsed Fas-deficient BMDCs can induce more severe allergic airway inflammation than HDM-pulsed wild-type BMDCs.

### Fas Signaling in BMDCs Does Not Affect CD4^+^ T Cell Proliferation, Apoptosis, and Activation *in vivo*

**Figure 2:**
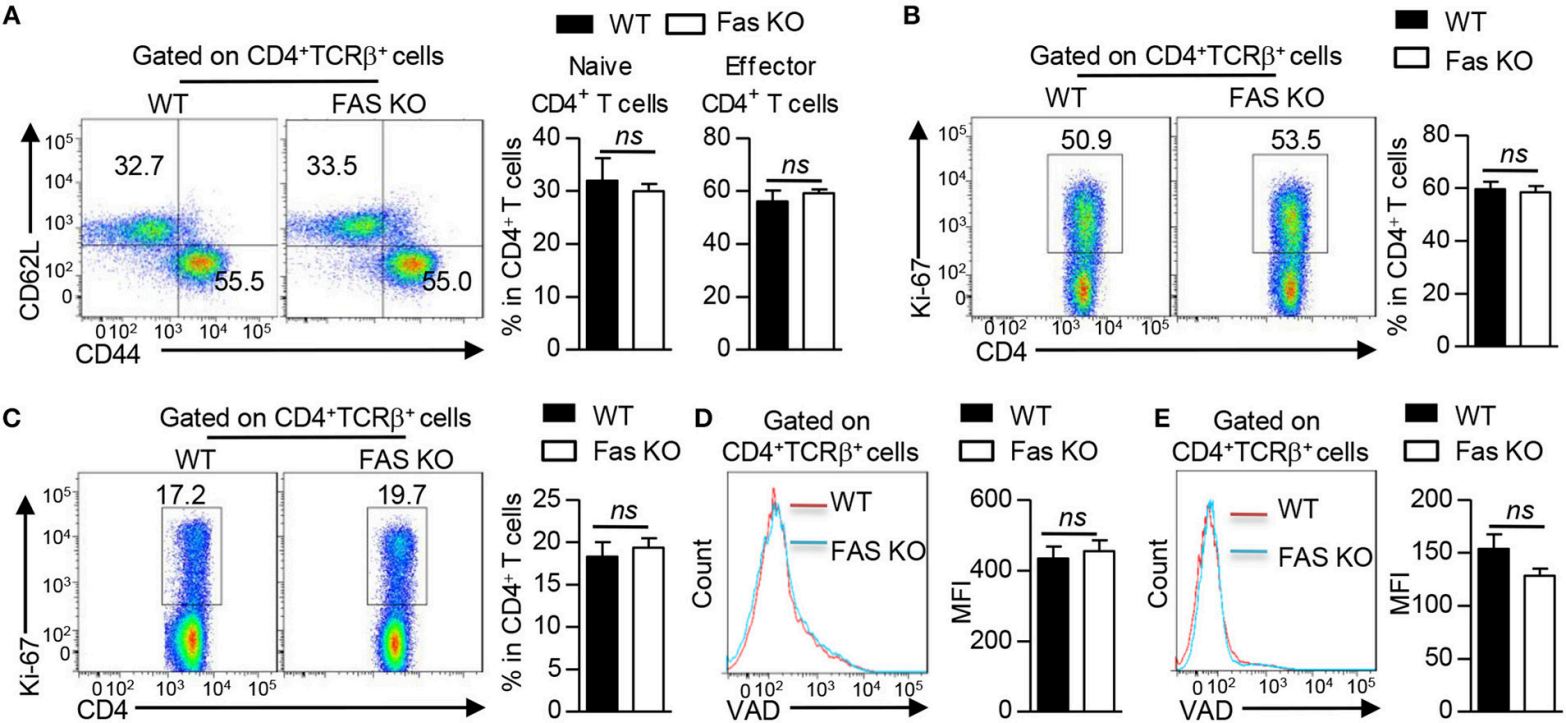
Fas signaling in BMDCs does not affect CD4^+^ T cell proliferation, apoptosis and activation *in vivo*. Mice were treated as Figure 1A described. **(A)** Flow cytometry (left) and proportions (right) of naïve CD4^+^ T cells (CD4^+^TCRβ^+^ CD62L^+^CD44^−^) and effector CD4^+^ T cells (CD4^+^TCRβ^+^CD62L^−^CD44^+^) in lung tissues. **(B,C)** Flow cytometry (left) and proportion (right) of Ki-67 expression in CD4^+^ T cells of lung tissues **(B)** and mLN **(C)**. **(D,E)** Flow cytometry (left) and Median Fluorescence Intensity (MFI) of VAD (right) staining in CD4^+^ T cells of lung tissues **(D)** and mLN **(E)**. *ns*, not significant. Data are representative of two independent experiments with 4–6 mice per group **(A–E)**. Student's *t*-tests **(A–E)** were performed and data were presented as mean ± SEM.

In addition to a role of innate immune cells in the development of allergic inflammation, the adaptive immune system also play an important role in driving and sustaining this inflammation (27). A comparable CD4^+^ T cell activation was observed in lung tissues of the recipients transferred with HDM-pulsed wild-type or Fas-deficient BMDCs (Figure 2A). We also examined whether Fas-deficient in BMDCs could affect CD4^+^ T cell proliferation or apoptosis *in vivo*, we performed Ki-67 and VAD staining assay, respectively. Our results showed that the proportion of Ki-67^+^CD4^+^ T cells and the median fluorescence intensity (MFI) value of VAD^+^CD4^+^ T cell in lung tissues and mediastinal lymph nodes (mLN) had no differences between the recipients transferred with HDM-pulsed wild-type BMDCs and those transferred with Fas-deficient BMDCs (Figures 2B–E). Taken together, these results show that Fas signaling in BMDCs is not required for CD4^+^ T cell activation, proliferation and apoptosis upon HDM treatment.

### Fas-Deficient BMDCs Promote Th2 Responses Upon HDM Treatment

**Figure 3:**
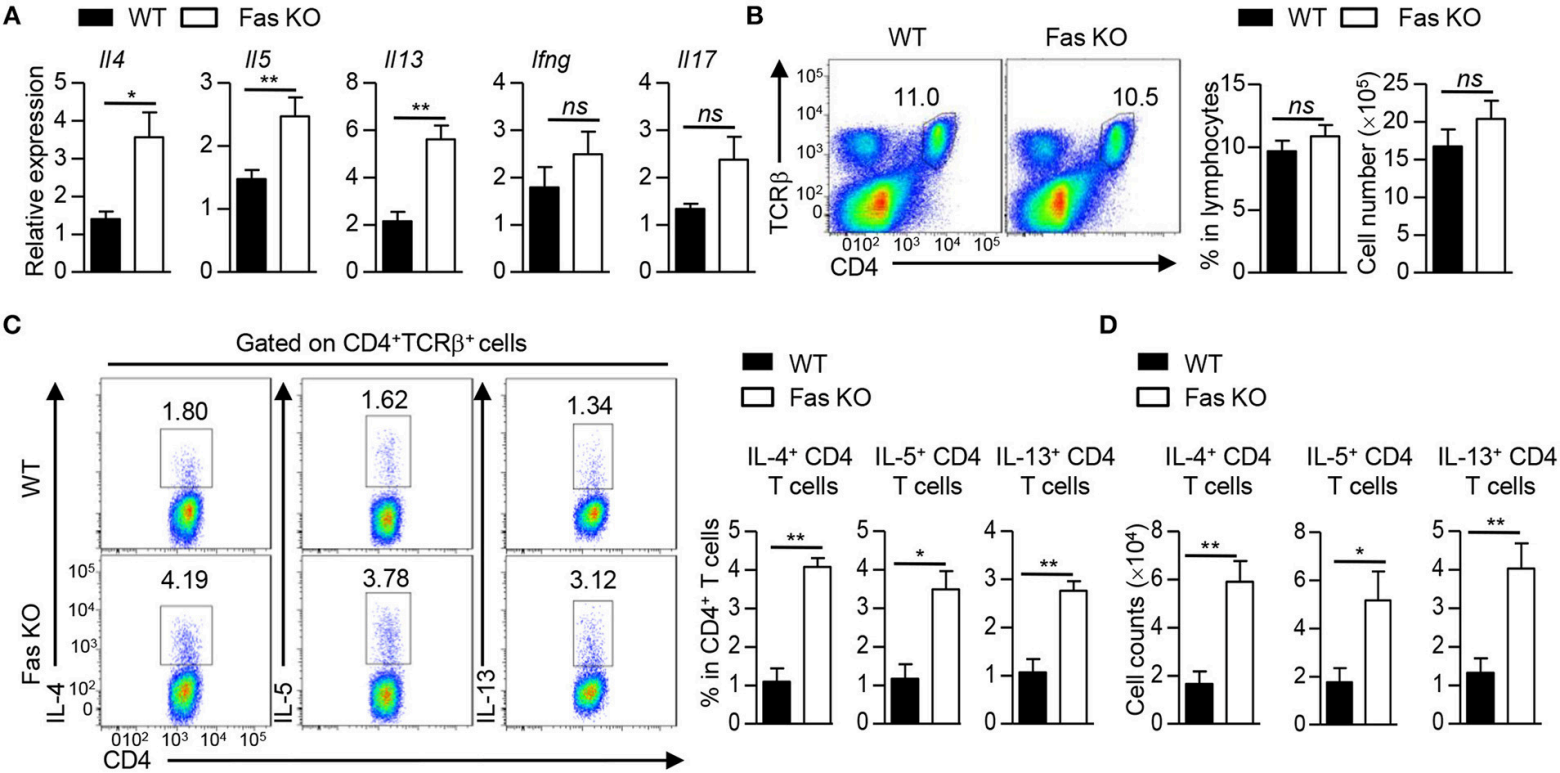
Fas-deficient BMDCs promote Th2 responses upon HDM treatment. Mice were treated as Figure 1A described. **(A)** mRNA expression of *Il4, Il5, Il13, Ifng*, and *Il17* in lung tissues was detected by qPCR and normalized to *Hprt*. **(B)** Flow cytometry (left), percentage (middle), and cell number (right) of CD4^+^ T cells in lung tissues. **(C)** Flow cytometry (left) and percentage of IL-4^+^, IL-5^+^, and IL-13^+^ CD4^+^ T cells in lung tissues. **(D)** Cell number of IL-4^+^, IL-5^+^, and IL-13^+^CD4^+^ T cells in lung tissues. ^*^P < 0.05, ^**^P < 0.01, *ns*, not significant. Data are representative of two independent experiments with 4–5 mice **(A–D)** per group. Student's *t*-tests **(A–D)** were performed and data were presented as mean ± SEM.

Given that there was no difference in the proliferation and apoptosis of CD4^+^ T cells in the recipients transferred with HDM-pulsed wild-type or Fas-deficient BMDCs, we reasoned that the difference in inflammatory response might be due to the potential of these cells to produce inflammatory cytokines. Thus, we first measured cytokine expression in lung tissues of these two group mice. The qPCR results showed that the recipients transferred with HDM-pulsed Fas-deficient BMDCs had higher mRNA expression of *Il4* (encoding IL-4), *Il5* (encoding IL-5), and *Il13* (encoding IL-13), but comparable expression of *Ifng* (encoding IFNγ) and *Il17a* (encoding IL-17A) in lung tissues than those transferred with HDM-pulsed wild-type BMDCs (Figure 3A). Although a comparable percentage and cell number of CD4^+^ T cells was observed in lung tissues of the two HDM-pulsed groups (Figure 3B), intracellular staining showed that recipients transferred with HDM-pulsed Fas-deficient BMDCs had higher percentage of IL-4^+^, IL-5^+^, and IL-13^+^CD4^+^ T cells in lung tissues than those transferred with wild-type BMDCs, along with higher cell number of IL-4^+^, IL-5^+^, and IL-13^+^CD4^+^ T cells (Figures 3C,D), whereas the percentage and cell number of IL-17^+^CD4^+^ T cells were similar in the two groups (Supplementary Figures 2A,B). However, the percentage and cell number of IL-4^+^, IL-5^+^, and IL-13^+^CD4^+^ T cells had no difference between the recipients transferred with un-pulsed wild-type and Fas-deficient BMDCs (Supplementary Figures 2C,D). Taken together, these results demonstrate that HDM-pulsed Fas-deficient BMDCs promote Th2 responses *in vivo*.

### Fas Signaling in BMDCs Instructs Th2 Cell Differentiation *in vitro*

**Figure 4:**
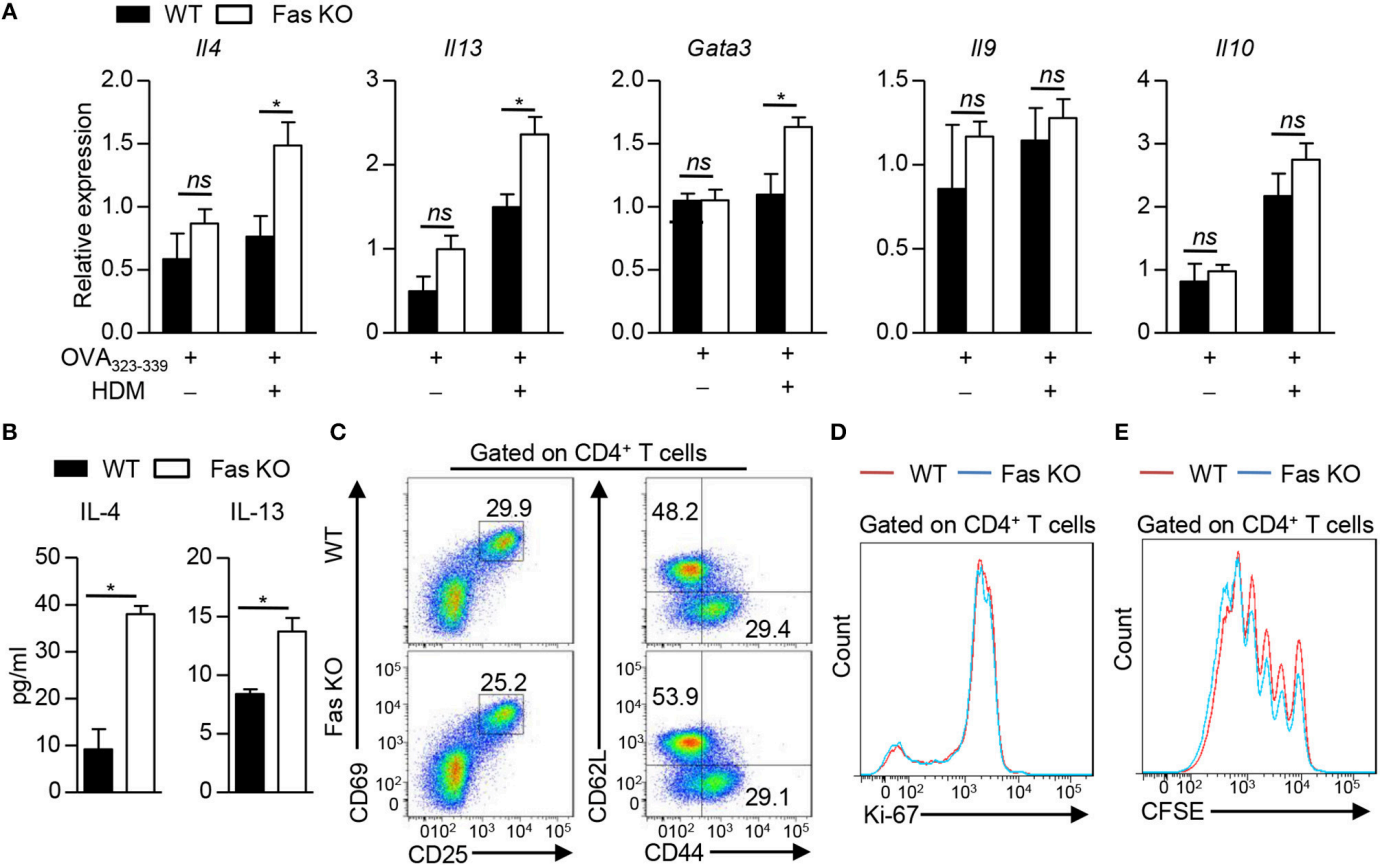
Fas signaling in BMDCs instructs Th2 cell differentiation *in vitro*. **(A–D)** Co-culture of wild-type and Fas-deficient BMDCs with naïve OT-II CD4^+^ T cells (CD4^+^CD25^−^CD62L^+^CD44^−^) in the presence of OVA_323−339_ with or without HDM for 48 h **(A)**, 3 days **(B,D)**, 18 h **(C)**, and then CD4^+^ T cells were harvested for analyses. **(A)** mRNA expression of *Il4, Il13, Gata3, Il9*, and *Il10* was detected by qPCR and normalized to *Hprt*. **(B)** Production of IL-4 and IL-13 was detected by ELISA. **(C)** Flow cytometry of the activation status of OT-II CD4^+^ T cells. **(D)** Flow cytometry of Ki-67 expression in OT-II CD4^+^ T cells. **(E)** Naïve OT-II CD4^+^ T cells were labeled with CFSE and then co-cultured with wild-type or Fas-deficient BMDCs in the presence of OVA_323−339_ and HDM for 4 days. Flow cytometry of CD4^+^ T cell proliferation. ^*^P < 0.05, *ns*, not significant. Data are representative of two independent experiments with duplicate or triplicate wells per group **(A**,**B)**. Student's *t*-tests were performed and data were presented as mean ± SEM.

To determine whether the increased Th2 response *in vivo* is due to the direct interaction between DCs and T cells, we co-cultured wild-type or Fas-deficient BMDCs with naïve OT-II CD4^+^ T cells in the presence of OVA_323−339_ peptide with or without HDM. The polarization of CD4^+^ T cells was determined by qPCR or ELISA, respectively. OT-II CD4^+^ T cells activated with HDM-stimulated Fas-deficient BMDCs had higher IL-4 and IL-13 expression both in mRNA and in protein levels than those stimulated with wild-type BMDCs (Figures 4A,B). GATA3, the master transcription factor for Th2 cell differentiation, was also found increased in T cells activated by HDM-pulsed Fas-deficient BMDCs, while the expression of *Il9* and *Il10* was comparable (Figure 4A). The activation and proliferation of OT-II CD4^+^ T cells activated by HDM-pulsed both wild-type and Fas-deficient BMDCs were comparable (Figures 4C–E). Collectively, these data indicate that Fas signaling mediates the direct crosstalk between BMDCs and Th2 cells upon HDM stimulation *in vitro*.

### Fas-Deficient BMDCs Promote Th2 Differentiation by Inhibiting IL-12 Expression

**Figure 5:**
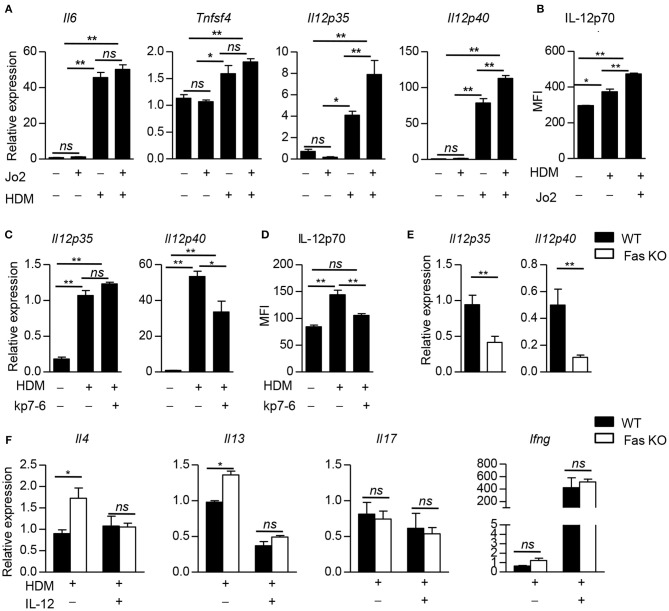
Fas-deficient BMDCs promote Th2 differentiation by inhibiting IL-12 expression. **(A)** Wild-type BMDCs were stimulated with Fas agonistic antibody Jo2, HDM, and HDM with Jo2 for 5 h, un-stimulated BMDCs as control. Expression of *Il6, Tnfsf4, Il12p35*, and *Il12p40*, was detected by qPCR and normalized to *Hprt*. **(B)** Wild-type BMDCs were stimulated with HDM in the presence or absence of Fas agonistic antibody Jo2 for 8 h and Golgistop was added into the system in the last 4 h. The MFI of IL-12 was detected by intracellular staining. **(C)** Wild-type BMDCs were stimulated with HDM in the presence or absence of Fas antagonistic antibody kp7-6 for 5 h, un-stimulated BMDCs as control. Expression *Il12p35* and *Il12p40* was detected by qPCR normalized to *Hprt*. **(D)** Wild-type BMDCs were stimulated with HDM in the presence or absence of Fas antagonistic antibody kp7-6 for 10 h and Golgistop was added into the system in the last 4 h, un-stimulated BMDCs as control. The MFI of IL-12 was detected by intracellular staining. **(E)** Wild-type and Fas-deficient BMDCs were stimulated with HDM and Fas agonistic antibody Jo2 for 5 h. mRNA expression of *Il12p35* and *Il12p40* was detected by qPCR and normalized to *Hprt*. **(F)** IL-12 (1 ng/ml) was added into the BMDC–OT-II CD4^+^ T cell co-culture system. mRNA expression of *Il4, Il13, Il17*, and *Ifng* in CD4^+^ T cells was detected by qPCR and normalized to *Hprt*. ^*^P < 0.05, ^**^P < 0.01, *ns*, not significant. Data are representative of two independent experiments with duplicate or triplicate wells per group **(A–F)**. Student's *t*-tests **(E)**, one-way ANOVA **(A–D)** or two-way ANOVA **(F)** were performed and data were presented as mean ± SEM.

Next we explored the molecular mechanism by which Fas signaling in BMDCs to shape Th2 differentiation upon HDM stimulation. We stimulated wild-type BMDCs with Fas agonistic antibody Jo2, the expression of *Cd86, Tslp, Il10, Il6*, and *Tnfsf4*, which had been reported to regulate Th2 polarization, had no difference compared to control group (Figure 5A and Supplementary Figure 3A). HDM stimulation could dramatically increase the expression of *Il6* and *Tnfsf4*, while Fas agonistic antibody Jo2 did not affect the expression of these two genes (Figure 5A), indicating that FAS signaling is not required for *Il6* and *Tnfsf4* expression in DCs upon HDM stimulation. IL-12 has been reported to affect Th2 differentiation (28). Fas agonistic antibody Jo2 stimulation could not affect the expression of *Il12p35* and *Il12p40* in wild-type BMDCs without HDM stimulation (Figure 5A). Upon HDM stimulation, the expression of *Il12p35* and *Il12p40* was significantly increased compared with non-HDM stimulation, and Fas agonistic antibody Jo2 could further enhance the expression of *Il12p35* and *Il12p40* in mRNA level and IL-12p70 in protein level (Figures 5A,B). Given that HDM-stimulated BMDCs had increased FasL, Fas antagonistic antibody kp7-6-treated HDM-pulsed wild-type BMDCs produced lower *Il12p40* in mRNA level and IL-12p70 in protein level than those of BMDCs stimulated with HDM alone, but the expression of *Il12p35* had no difference between HDM and HDM plus kp7-6 stimulated BMDCs (Figures 5C,D and Supplementary Figure 3B). Fas-deficient BMDCs had decreased expression of *Il12p35* and *Il12p40* compared to wild-type BMDCs upon HDM stimulation in the presence of Fas agonistic antibody Jo2 (Figure 5E). We next examined whether the lower expression of IL-12 in Fas-deficient BMDCs could contribute to the increased Th2 differentiation. We added exogenous IL-12 into the BMDC–T cell co-culture system and found that the IL-12 supplement could significantly decrease the expression of Th2-related cytokines, such as IL-4 and IL-13 in OT-II CD4^+^ T cells activated by Fas-deficient BMDCs. The expression of IFNγ and IL-17 was comparable between wild-type and Fas-deficient BMDC activated T cells (Figure 5F). Altogether, these results indicate that Fas-deficient BMDCs promote Th2 differentiation through downregulation of IL-12 expression.

### Fas Signaling Regulates IL-12 Expression by Modulation of ERK Activation in BMDCs

**Figure 6:**
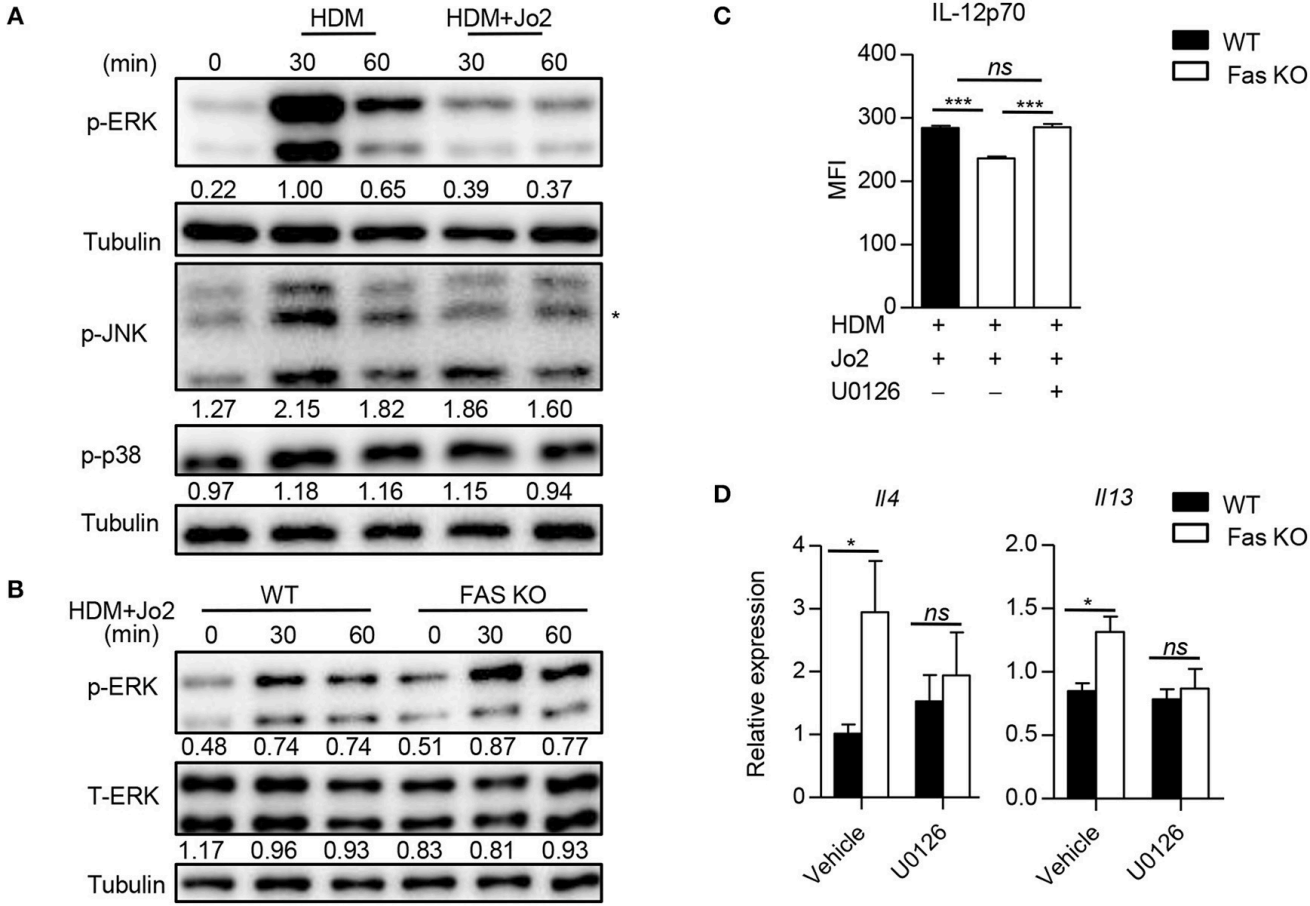
Fas signaling regulates IL-12 expression by modulation of ERK activation in BMDCs. **(A)** Activity of ERK, JNK, and p38 in wild-type BMDCs stimulated with HDM in the presence or absence of Fas agonistic antibody Jo2 was detected by western blot. Asterisk indicates non-specific bands. Numbers under the lanes indicate band intensity related to that of alpha tubulin. **(B)** Activation of ERK in wild-type and Fas-deficient BMDCs stimulated with HDM plus Fas agonistic antibody Jo2. Numbers under the lanes indicate band intensity related to that of alpha tubulin. **(C)** Wild-type and Fas-deficient BMDCs were pre-treated with U0126 for 30 min and then stimulated with HDM and Fas agonistic antibody Jo2 for 8 h. Golgistop was added into the system in the last 4 h. The MFI of IL-12 was detected by intracellular staining. **(D)** Wild-type and Fas-deficient BMDCs were pre-treated with U0126 for 30 min and then stimulated with HDM for 5 h, washed and co-cultured with OT-II CD4^+^ T cells for 48 h. Expression of *Il4* and *Il13* was detected by qPCR and normalized to *Hprt*. ^*^P < 0.05, ^***^P < 0.001, *ns*, not significant. Data are representative of two independent experiments with duplicate or triplicate wells per group **(C)** or combination of two experiments with consistent results **(D)**. One-way ANOVA **(C)** or two-way ANOVA **(D)** was performed and data were presented as mean ± SEM.

We next examined the downstream signaling of Fas involved in the regulation of IL-12 expression by analyzing the activation of p38, JNK, and ERK in BMDCs. We found that the phosphorylation of ERK was decreased in wild-type BMDCs treated with Fas agonistic antibody Jo2 and HDM compared to those treated with HDM alone (Figure 6A). Accordingly, the activation of ERK in Fas-deficient BMDCs was increased compared to that of wild-type BMDCs upon HDM and Fas agonistic antibody Jo2 stimulation (Figure 6B). To determine whether the increased ERK activation was contributed to the decreased IL-12 expression in Fas-deficient BMDCs, we treated Fas-deficient BMDCs with specific ERK inhibitor U0126, which resulted in a dramatically increased IL-12p70 expression in Fas-deficient BMDCs (Figure 6C). Consequently, U0126-treated Fas-deficient BMDCs completely restored the increased IL-4 and IL-13 expression in T cells to the level of T cells activated with wild-type BMDCs (Figure 6D). These results indicate that Fas signaling regulates IL-12 expression and Th2 differentiation through modulating the ERK activity in BMDCs.

## Discussion

Numerous studies have reported that administration of allergen-pulsed DCs is sufficient to induce airway inflammation by polarizing Th2 responses (3, 29–31). However, the mechanism of DCs to regulate Th2 cell differentiation is still unclear (32). In this study, we used a BMDC-transfer protocol to investigate the important role of DC-specific Fas signaling in the pathogenesis of HDM-induced Th2-mediated allergic inflammation. We found that HDM-pulsed Fas-deficient BMDCs could promote Th2 responses and allergic eosinophilic inflammation, without affecting T cell apoptosis and proliferation in the recipients. Our study identified a crucial role of Fas signaling in regulating IL-12 expression by modulating ERK activity in DCs to direct Th2 differentiation upon HDM stimulation, which may provide an attractive treatment strategy for allergic diseases.

DCs constitutively express non-canonical costimulatory molecule Fas (13), which is activated by FasL or Fas agonistic antibody Jo2 to induce an apoptotic signaling. Numerous studies have shown that activated Fas signaling in DCs induces the secretion of cytokines such as IL-1β and CXC or CC chemokines (13, 33, 34), which may play important roles in the recruitment, activation and proliferation of naïve T cells (14, 35). Fas–FasL interaction on T cells has been proposed to promote the differentiation of naïve T cells into functional T cells (14), but little is known about how the Fas signaling in DCs regulating Th2 differentiation. DCs could promote Th2 differentiation through upregulation signal 2 (such as CD86, OX40L) (7, 36, 37) or signal 3 (such as IL-6, IL-10,TSLP) (38–40). In this study, we found that Fas agonistic antibody Jo2 stimulation did not affect the expression of OX40L and IL-6. However, Jo2 stimulation increased IL-12 expression in BMDCs stimulated with HDM. Accordingly, the ablation of Fas in DCs largely reduced the expression of IL-12, which contributed to the increased Th2 differentiation. Our results identify a new mechanism by which DC regulating Th2 responses through modulation of IL-12 production during inflammation development.

ERK signaling has been shown to play important roles in Fas-mediated non-apoptotic function. Ligation of Fas agonistic antibody Jo2 with Fas on DCs can promote the activation of ERK and subsequent IL-1β secretion (34). In the current study, we found that Jo2 stimulation could dramatically decrease the activity of ERK in the presence of HDM. This different role might be caused by the type of stimuli and the status of the BMDCs, and further study need to be explored the potential mechanism for this different regulation by ERK. ERK signaling could profoundly influence the immune response of T cells. ERK activity in CD4^+^ T cells has a key role in Th2 cell polarization (41). Blocking MEK-ERK signaling effectively suppresses IL-12p40 production from *Neospora caninum* infected peritoneal macrophages (42). In contrast, ERK signaling also has been reported to be an important negative regulator of IL-12 secretion in cigarette smoke extract (CSE) stimulated DCs (43). In this study, we found that a higher ERK activity could suppress IL-12 production in HDM-stimulated Fas-deficient BMDCs. Taken together, this study uncovers a specific role of Fas signaling in BMDCs in the regulation of Th2 differentiation and Th2-mediated allergic inflammation. Modulation of Fas signaling on DCs may provide a new strategy for treatment of allergic diseases.

## Author Contributions

MH designed and performed the *in vivo* and cellular experiments and contributed to manuscript writing. RH performed the *in vitro* and cellular experiments. JM contributed mouse models. CC contributed to animal colony management. BZ contributed to western blot analysis. HL and JY provided reagents. GH designed experiments, analyzed the data, wrote the manuscript, and provided overall directions.

### Conflict of Interest Statement

The authors declare that the research was conducted in the absence of any commercial or financial relationships that could be construed as a potential conflict of interest.

## References

[1] Lambrecht BN Hammad H. The immunology of asthma. Nat Immunol. (2015) 16:45–56. 10.1038/ni.3049 25521684

[2] van Helden MJ Lambrecht BN. Dendritic cells in asthma. Curr Opin Immunol. (2013) 25:745–54. 10.1016/j.coi.2013.10.002 24455765

[3] Chuang YH Suen JL Chiang BL. Fas-ligand-expressing adenovirus-transfected dendritic cells decrease allergen-specific T cells and airway inflammation in a murine model of asthma. J Mol Med. (2006) 84:595–603. 10.1007/s00109-006-0047-3 16565865

[4] Gregory LG Lloyd CM. Orchestrating house dust mite-associated allergy in the lung. Trends Immunol. (2011) 32:402–11. 10.1016/j.it.2011.06.006 21783420 PMC3381841

[5] Tjota MY Hrusch CL Blaine KM Williams JW Barrett NA Sperling AI Signaling through FcR gamma-associated receptors on dendritic cells drives IL-33-dependent T(H)2-type responses. J Allergy Clin Immun. (2014) 134:706–13.e8. 10.1016/j.jaci.2014.06.013 25088053 PMC4149927

[6] Mayer A Debuisson D Denanglaire S Eddahri F Fievez L Hercor M. Antigen presenting cell-derived IL-6 restricts Th2-cell differentiation. Eur J Immunol. (2014) 44:3252–62. 10.1002/eji.201444646 25092208

[7] Krishnamoorthy N Oriss TB Paglia M Fei M Yarlagadda M Vanhaesebroeck B. Activation of c-Kit in dendritic cells regulates T helper cell differentiation and allergic asthma. Nat Med. (2008) 14:565–73. 10.1038/nm1766 18454155 PMC3664066

[8] Ito T Wang YH Duramad O Hori T Delespesse GJ Watanabe N. TSLP-activated dendritic cells induce an inflammatory T helper type 2 cell response through OX40 ligand. J Exp Med. (2005) 202:1213–23. 10.1084/Jem.20051135 16275760 PMC2213234

[9] Lambrecht BN Hammad H. Biology of lung dendritic cells at the origin of asthma. Immunity (2009) 31:412–24. 10.1016/j.immuni.2009.08.008 19766084

[10] Shibaki A Katz SI. Activation through CD40 ligation induces functional Fas ligand expression by Langerhans cells. Eur J Immunol. (2001) 31:3006–15. 10.1002/1521-4141(2001010)31:10<3006::AID-IMMU3006>3.0.CO;2-L 11592077

[11] Duez C Tomkinson A Shultz LD Bratton DL Gelfand EW. Fas deficiency delays the resolution of airway hyperresponsiveness after allergen sensitization and challenge. J Allergy Clin Immunol. (2001) 108:547–56. 10.1067/mai.2001.118288 11590380

[12] Tong J Bandulwala HS Clay BS Anders RA Shilling RA Balachandran DD. Fas-positive T cells regulate the resolution of airway inflammation in a murine model of asthma. J Exp Med. (2006) 203:1173–84. 10.1084/jem.20051680 16618792 PMC2121201

[13] Rescigno M Piguet V Valzasina B Lens S Zubler R French L. Fas engagement induces the maturation of dendritic cells (DCs), the release of interleukin (IL)-1beta, and the production of interferon gamma in the absence of IL-12 during DC-T cell cognate interaction: a new role for Fas ligand in inflammatory responses. J Exp Med. (2000) 192:1661–8. 10.1084/jem.192.11.1661 11104808 PMC2193091

[14] Meyer Zu Horste G Przybylski D Schramm MA Wang C Schnell A Lee Y. Fas promotes T Helper 17 cell differentiation and inhibits T Helper 1 cell development by binding and Sequestering Transcription Factor STAT1. Immunity (2018) 48:556–69 e7. 10.1016/j.immuni.2018.03.008 29562202

[15] Bien K Zmigrodzka M Orlowski P Fruba A Szymanski L Stankiewicz W. Involvement of Fas/FasL pathway in the murine model of atopic dermatitis. Inflamm Res. (2017) 66:679–90. 10.1007/s00011-017-1049-z 28434120 PMC5501908

[16] Stewart SA Dykxhoorn DM Palliser D Mizuno H Yu EY An DS. Lentivirus-delivered stable gene silencing by RNAi in primary cells. RNA (2003) 9:493–501. 10.1261/rna.2192803 12649500 PMC1370415

[17] Harris NL Watt V Ronchese F Le Gros G. Differential T cell function and fate in lymph node and nonlymphoid tissues. J Exp Med. (2002) 195:317–26. 10.1084/jem.20011558 11828006 PMC2193599

[18] Ji WJ Ma YQ Zhou X Zhang YD Lu RY Sun HY. Temporal and spatial characterization of mononuclear phagocytes in circulating, lung alveolar and interstitial compartments in a mouse model of bleomycin-induced pulmonary injury. J Immunol Methods (2014) 403:7–16. 10.1016/j.jim.2013.11.012 24280595

[19] Fulkerson PC Fischetti CA Hassman LM Nikolaidis NM Rothenberg ME. Persistent effects induced by IL-13 in the lung. Am J Respir Cell Mol Biol. (2006) 35:337–46. 10.1165/rcmb.2005-0474OC 16645178 PMC2643287

[20] Kang Z Swaidani S Yin W Wang C Barlow JL Gulen MF. Epithelial cell-specific Act1 adaptor mediates interleukin-25-dependent helminth expulsion through expansion of Lin(-)c-Kit(+) innate cell population. Immunity (2012) 36:821–33. 10.1016/j.immuni.2012.03.021 22608496 PMC3376903

[21] Zheng W Flavell RA. The transcription factor GATA-3 is necessary and sufficient for Th2 cytokine gene expression in CD4 T cells. Cell (1997) 89:587–96. 9160750 10.1016/s0092-8674(00)80240-8

[22] Angkasekwinai P Chang SH Thapa M Watarai H Dong C. Regulation of IL-9 expression by IL-25 signaling. Nat Immunol. (2010) 11:250–6. 10.1038/ni.1846 20154671 PMC2827302

[23] Slawek A Maj T Chelmonska-Soyta A. CD40, CD80, and CD86 costimulatory molecules are differentially expressed on murine splenic antigen-presenting cells during the pre-implantation period of pregnancy, and they modulate regulatory T-cell abundance, peripheral cytokine response, and pregnancy outcome. Am J Reprod Immunol. (2013) 70:116–26. 10.1111/aji.12108 23445188

[24] Bell BD Kitajima M Larson RP Stoklasek TA Dang K Sakamoto K The transcription factor STAT5 is critical in dendritic cells for the development of TH2 but not TH1 responses. Nat Immunol. (2013) 14:364–71. 10.1038/ni.2541 23435120 PMC4161284

[25] Huang G Wang Y Vogel P Kanneganti TD Otsu K Chi H. Signaling via the kinase p38alpha programs dendritic cells to drive TH17 differentiation and autoimmune inflammation. Nat Immunol. (2012) 13:152–61. 10.1038/ni.2207 22231518 PMC3262925

[26] Mesnil C Raulier S Paulissen G Xiao X Birrell MA Pirottin D. Lung-resident eosinophils represent a distinct regulatory eosinophil subset. J Clin Invest. (2016) 126:3279–95. 10.1172/JCI85664 27548519 PMC5004964

[27] Holgate ST. Innate and adaptive immune responses in asthma. Nat Med. (2012) 18:673–83. 10.1038/nm.2731 22561831

[28] Lamhamedi-Cherradi SE Martin RE Ito T Kheradmand F Corry DB Liu YJ. Fungal proteases induce Th2 polarization through limited dendritic cell maturation and reduced production of IL-12. J Immunol. (2008) 180:6000–9. 10.4049/jimmunol.180.9.6000 18424720 PMC3652558

[29] Lambrecht BN De Veerman M Coyle AJ Gutierrez-Ramos JC Thielemans K Pauwels RA. Myeloid dendritic cells induce Th2 responses to inhaled antigen, leading to eosinophilic airway inflammation. J Clin Invest. (2000) 106:551–9. 10.1172/JCI8107 10953030 PMC380243

[30] Shao Z Bharadwaj AS McGee HS Makinde TO Agrawal DK. Fms-like tyrosine kinase 3 ligand increases a lung DC subset with regulatory properties in allergic airway inflammation. J Allergy Clin Immunol. (2009) 123:917–24.e2. 10.1016/j.jaci.2009.01.052 19348927 PMC2690643

[31] Takagi T Taguchi O Toda M Ruiz DB Bernabe PG D'Alessandro-Gabazza CN. Inhibition of allergic bronchial asthma by Thrombomodulin is mediated by dendritic cells. Am J Respir Critic Med. (2011) 183:31–42. 10.1164/rccm.201001-0107OC 20709825

[32] Na H Cho M Chung Y. Regulation of Th2 cell immunity by dendritic cells. Immune Network (2016) 16:1–12. 10.4110/in.2016.16.1.1 26937227 PMC4770095

[33] Guo Z Zhang M Tang H Cao X. Fas signal links innate and adaptive immunity by promoting dendritic-cell secretion of CC and CXC chemokines. Blood (2005) 106:2033–41. 10.1182/blood-2004-12-4831 15941911

[34] Guo Z Zhang M An H Chen W Liu S Guo J. Fas ligation induces IL-1beta-dependent maturation and IL-1beta-independent survival of dendritic cells: different roles of ERK and NF-kappaB signaling pathways. Blood (2003) 102:4441–7. 10.1182/blood-2002-11-3420 12920043

[35] Kataoka T Budd RC Holler N Thome M Martinon F Irmler M. The caspase-8 inhibitor FLIP promotes activation of NF-kappaB and Erk signaling pathways. Curr Biol. (2000) 10:640–8. 10.1016/S0960-9822(00)00512-1 10837247

[36] van Rijt LS Vos N Willart M Kleinjan A Coyle AJ Hoogsteden HC Essential role of dendritic cell CD80/CD86 costimulation in the induction, but not reactivation, of TH2 effector responses in a mouse model of asthma. J Allergy Clin Immunol. (2004) 114:166–73. 10.1016/j.jaci.2004.03.044 15241361

[37] Wu Q Tang Y Hu X Wang Q Lei W Zhou L. Regulation of Th1/Th2 balance through OX40/OX40L signalling by glycyrrhizic acid in a murine model of asthma. Respirology (2016) 21:102–11. 10.1111/resp.12655 26467500

[38] Lin YL Chen SH Wang JY. Critical role of IL-6 in dendritic cell-induced allergic inflammation of asthma. J Mol Med. (2015) 94:51–9. 10.1007/s00109-015-1325-8 26232935

[39] Laouini D Alenius H Bryce P Oettgen H Tsitsikov E Geha RS. IL-10 is critical for Th2 responses in a murine model of allergic dermatitis. J Clin Invest. (2003) 112:1058–66. 10.1172/Jci200318246 14523043 PMC198527

[40] Zhang YL Zhou XP Zhou BH. DC-derived TSLP promotes Th2 polarization in LPS-primed allergic airway inflammation. Eur J Immunol. (2012) 42:1735–43. 10.1002/eji.201142123 22585305 PMC3662367

[41] Tripathi P Sahoo N Ullah U Kallionpaa H Suneja A Lahesmaa R. A novel mechanism for ERK-dependent regulation of IL4 transcription during human Th2-cell differentiation. Immunol Cell Biol. (2012) 90:676–87. 10.1038/icb.2011.87 21989417 PMC3419974

[42] Jin XX Gong PT Zhang XC Li GJ Zhu T Zhang MG. Activation of ERK signaling via TLR11 induces IL-12p40 production in peritoneal macrophages challenged by neospora caninum. Front Microbiol. (2017) 8:1393. 10.3389/fmicb.2017.01393 28798732 PMC5527353

[43] Kroening PR Barnes TW Pease L Limper A Kita H Vassall R. Cigarette smoke-induced oxidative stress suppresses generation of dendritic cell IL-12 and IL-23 through ERK-dependent pathways. J Immunol. (2008) 181:1536–47. 10.4049/jimmunol.181.2.1536 18606709 PMC2819390

