# Supplementary material for: Fas Signaling in Dendritic Cells Mediates Th2 Polarization in HDM-Induced Allergic Pulmonary Inflammation
Source: Front Immunol. 2018 Dec 21;9:3045. doi: 10.3389/fimmu.2018.03045 (PMC6308134; doi:10.3389/fimmu.2018.03045)
Supplement: Supplementary file 1 [file Presentation_1.PPTX]

## Slide 1
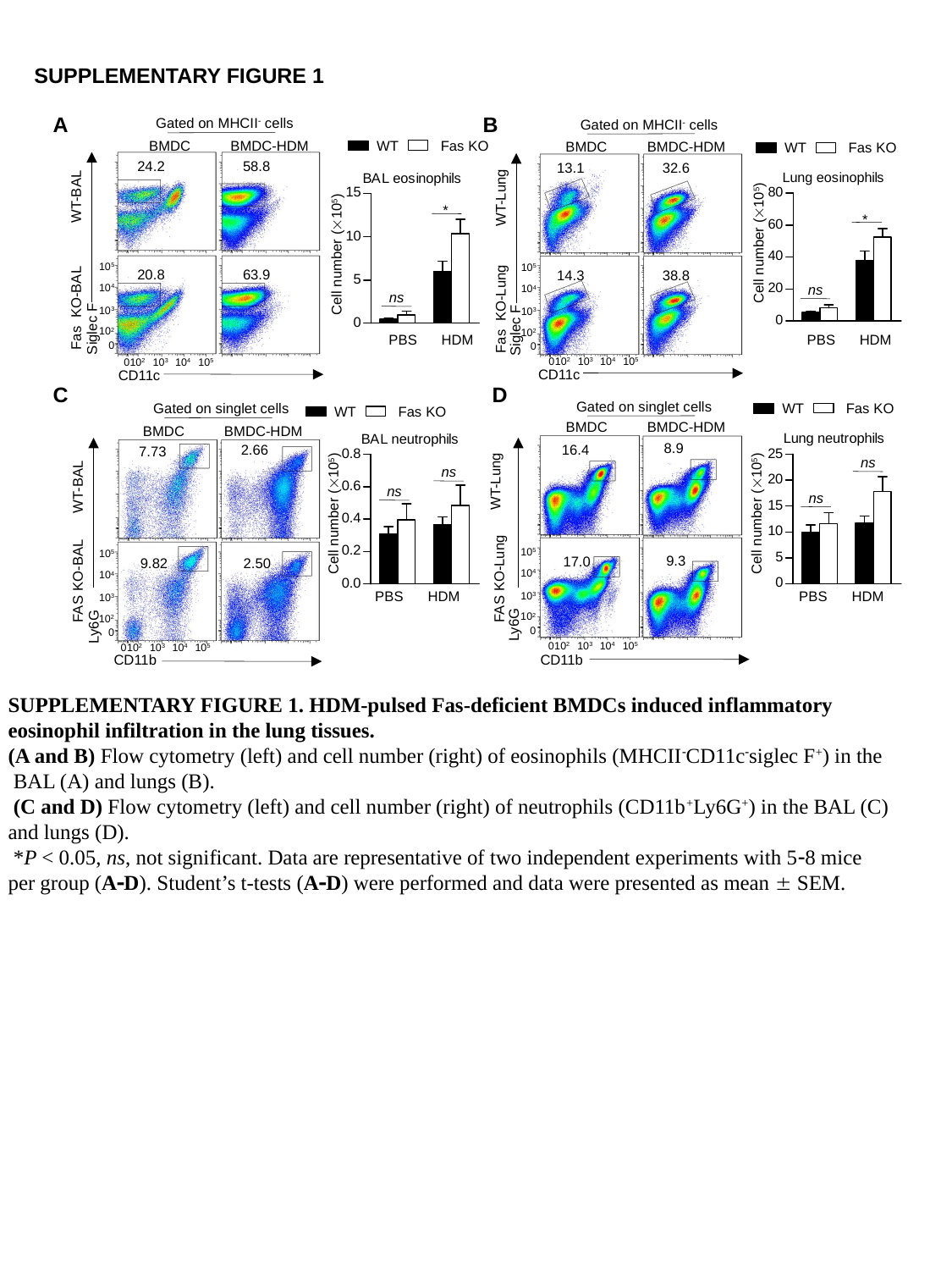

SUPPLEMENTARY FIGURE 1
A
B
Gated on MHCII− cells
Gated on MHCII− cells
WT
Fas KO
BMDC
BMDC-HDM
BMDC
BMDC-HDM
WT
Fas KO
24.2
58.8
13.1
32.6
WT-BAL
WT-Lung
*
*
Cell number (×105)
Cell number (×105)
105
104
103
102
0
105
104
103
102
0
20.8
63.9
14.3
38.8
ns
ns
Fas KO-BAL
Fas KO-Lung
Siglec F
Siglec F
PBS
HDM
PBS
HDM
0
102
103
104
105
0
102
103
104
105
CD11c
CD11c
C
D
Gated on singlet cells
WT
Fas KO
Gated on singlet cells
WT
Fas KO
BMDC
BMDC-HDM
BMDC
BMDC-HDM
8.9
16.4
2.66
7.73
ns
ns
WT-Lung
WT-BAL
ns
ns
Cell number (×105)
Cell number (×105)
105
104
103
102
0
105
104
103
102
0
9.3
17.0
2.50
9.82
FAS KO-Lung
FAS KO-BAL
PBS
HDM
PBS
HDM
Ly6G
Ly6G
0
102
103
104
105
0
102
103
104
105
CD11b
CD11b
SUPPLEMENTARY FIGURE 1. HDM-pulsed Fas-deficient BMDCs induced inflammatory
eosinophil infiltration in the lung tissues.
(A and B) Flow cytometry (left) and cell number (right) of eosinophils (MHCII−CD11c−siglec F+) in the
 BAL (A) and lungs (B).
 (C and D) Flow cytometry (left) and cell number (right) of neutrophils (CD11b+Ly6G+) in the BAL (C)
and lungs (D).
 *P < 0.05, ns, not significant. Data are representative of two independent experiments with 5−8 mice
per group (A−D). Student’s t-tests (A−D) were performed and data were presented as mean ± SEM.

## Slide 2
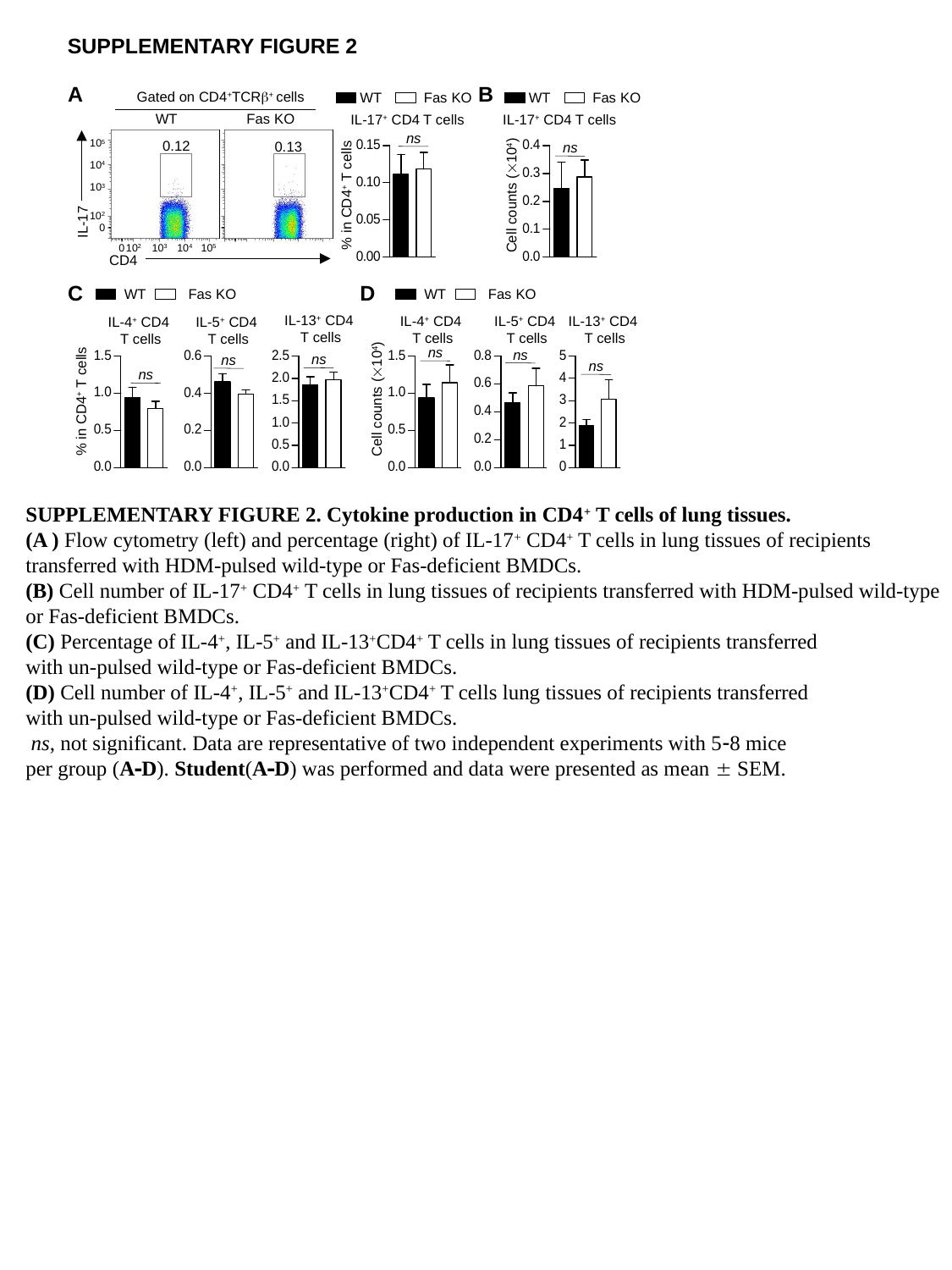

SUPPLEMENTARY FIGURE 2
A
B
Gated on CD4+TCRβ+ cells
WT
Fas KO
WT
Fas KO
WT
Fas KO
IL-17+ CD4 T cells
IL-17+ CD4 T cells
ns
105
104
103
102
0
0.12
0.13
ns
% in CD4+ T cells
Cell counts (×104)
IL-17
0
102
103
104
105
CD4
C
D
WT
Fas KO
WT
Fas KO
IL-13+ CD4
T cells
IL-4+ CD4
T cells
IL-5+ CD4
 T cells
IL-13+ CD4
 T cells
IL-4+ CD4
 T cells
IL-5+ CD4
T cells
ns
ns
ns
ns
ns
ns
Cell counts (×104)
% in CD4+ T cells
SUPPLEMENTARY FIGURE 2. Cytokine production in CD4+ T cells of lung tissues.
(A ) Flow cytometry (left) and percentage (right) of IL-17+ CD4+ T cells in lung tissues of recipients
transferred with HDM-pulsed wild-type or Fas-deficient BMDCs.
(B) Cell number of IL-17+ CD4+ T cells in lung tissues of recipients transferred with HDM-pulsed wild-type
or Fas-deficient BMDCs.
(C) Percentage of IL-4+, IL-5+ and IL-13+CD4+ T cells in lung tissues of recipients transferred
with un-pulsed wild-type or Fas-deficient BMDCs.
(D) Cell number of IL-4+, IL-5+ and IL-13+CD4+ T cells lung tissues of recipients transferred
with un-pulsed wild-type or Fas-deficient BMDCs.
 ns, not significant. Data are representative of two independent experiments with 5−8 mice
per group (A−D). Student(A−D) was performed and data were presented as mean ± SEM.

## Slide 3
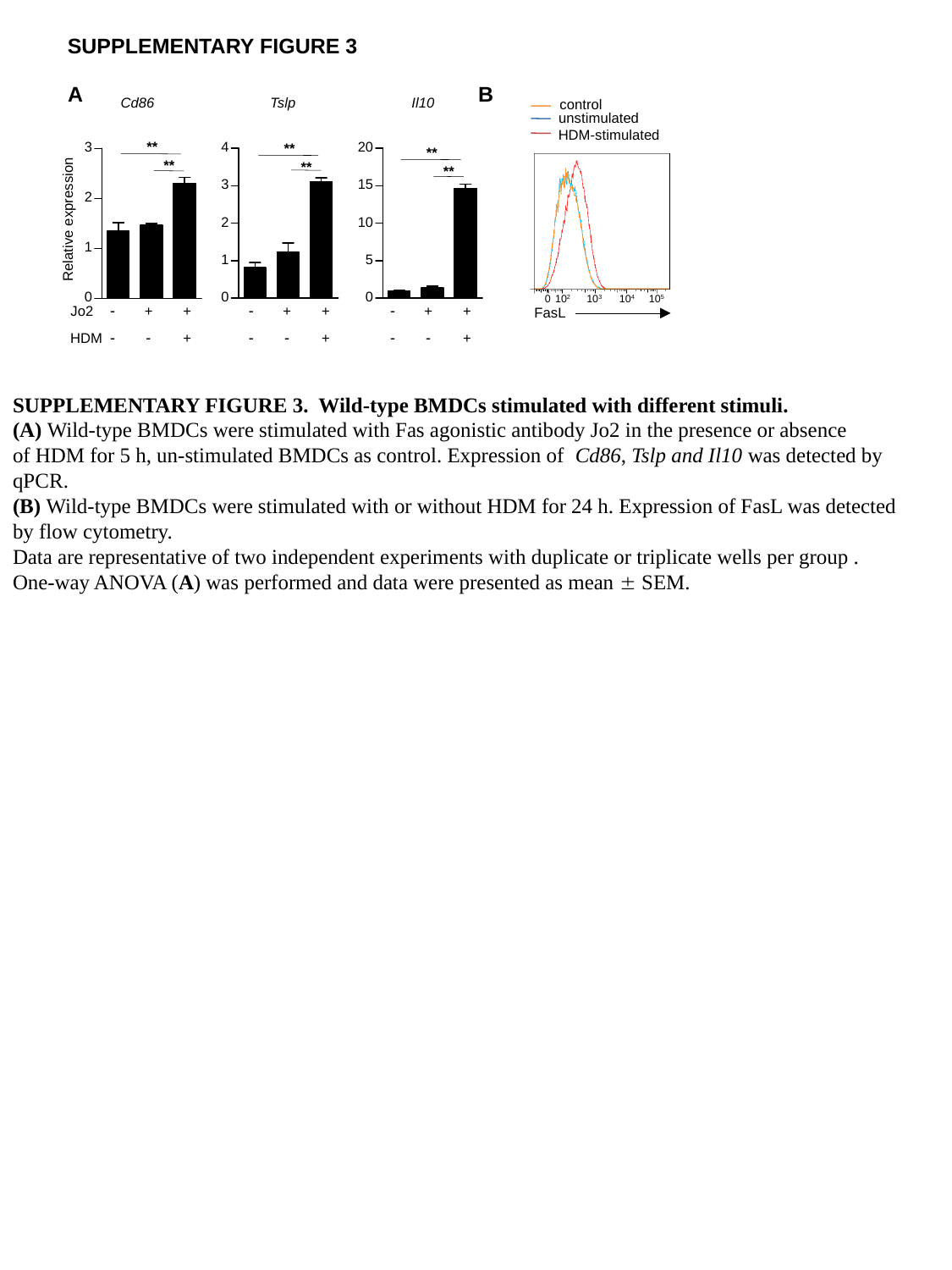

SUPPLEMENTARY FIGURE 3
A
B
Cd86
Tslp
Il10
control
unstimulated
HDM-stimulated
**
**
**
**
**
**
Relative expression
0
102
103
104
105
Jo2
−
+
+
−
−
+
−
+
+
−
−
+
−
+
+
−
−
+
FasL
HDM
SUPPLEMENTARY FIGURE 3. Wild-type BMDCs stimulated with different stimuli.
(A) Wild-type BMDCs were stimulated with Fas agonistic antibody Jo2 in the presence or absence
of HDM for 5 h, un-stimulated BMDCs as control. Expression of Cd86, Tslp and Il10 was detected by
qPCR.
(B) Wild-type BMDCs were stimulated with or without HDM for 24 h. Expression of FasL was detected
by flow cytometry.
Data are representative of two independent experiments with duplicate or triplicate wells per group .
One-way ANOVA (A) was performed and data were presented as mean ± SEM.
